# Accelerometer-based assessment of occupational standing time and its association with venous disorders – results of a cross-sectional field study

**DOI:** 10.1038/s41598-026-38327-8

**Published:** 2026-02-23

**Authors:** Jana Soeder, Carmen Volk, Luis Ulmer, Florestan Wagenblast, Robert Seibt, Erika Mendoza, Nicole Bott, Monika A. Rieger, Benjamin Steinhilber

**Affiliations:** 1 Institute of Occupational and Social Medicine and Health Services Research, University Hospital Tübingen, Tübingen, Germany; 2 Institute of Sports and Sports Science, Karlsruhe Institute of Technology, Karlsruhe, Germany; 3 Venenpraxis, Wunstorf, Germany

**Keywords:** Chronic venous diseases, Constrained posture, Standing work, Varicose veins, Venous reflux, Wearable, Medical research, Risk factors, Vascular diseases

## Abstract

Pathophysiologically, prolonged standing is associated with varicose veins (VV) or pathological venous reflux. Prior work-related epidemiological studies are inconclusive due to crude and imprecise exposure assessments. This cross-sectional field study explored the association between work-related standing time and VV or pathological reflux, assessed using wearables. Daily standing time was tracked by accelerometer and categorized: ≤2 vs. >2 but ≤4 vs. >4 h. Long-term cumulative standing exposure were estimated by combining accelerometer and survey data. Employees underwent CEAP classification and duplex ultrasound to measure lower leg reflux. The associations between standing time and other predictors on VV or reflux were analyzed by multiple logistic regression. 198 employees working ≥30 h and ≥4 days per week for ≥2y in logistics, trade, retail, manufacturing, healthcare, research, and service sectors were included (*n* = 116♀, Ø-age = 40 ± 9y). 18% (*n* = 36) stood ≤2 h, 46% (*n* = 91) >2 but ≤4 h, 36% (*n* = 71) >4 h per day. Long-term cumulative standing exposure was 10,145.0 ± 8,396.0 h. 24% (*n* = 47) were described with VV, 38% (*n* = 74) with reflux. While neither daily nor long-term cumulative standing time were significantly associated with VV or reflux, age and family history were. Our findings partly contradict previous epidemiological studies, highlighting the value of objectively measured activity patterns for future research.

**Supplementary Information:**

The online version contains supplementary material available at 10.1038/s41598-026-38327-8.

## Introduction

Varicose veins (VV) are a relatively common health issue, as indicated by previous epidemiological studies reporting prevalence rates ranging between 11.1 and 21.0% for lower-limb varicose veins among adults in Germany or Western Europe^1–3^. VV are defined as dilated palpable subcutaneous veins with a diameter of ≥3 mm^4^. VV develop when blood from the deep veins circulates back down the leg, causing the superficial veins to become overfilled, twisted and enlarged. Chronic venous disorders (CVD) due to VV typically start with symptoms such as oedema but can progress, and additional skin changes such as pigmentation or venous ulcers can occur^5^. CVD can be assessed using the CEAP classification (Clinical, Etiologic, Anatomic, Pathophysiologic), where VV are defined as C_2_ and chronic venous insufficiency (CVI) is defined as ≥C_3_ (C-aspect ranges from C_0_ to C_6_ and increases with severity)^6^. The CEAP classification also includes other reasons of CVI such as post-thrombotic changes, which will not be discussed here. Non-modifiable risk factors such as age^7^, family history^7^, female sex, and previous pregnancy^8^, as well as modifiable risk factors such as obesity^9^, smoking behavior, and alcohol consumption^10^ were shown to be associated with an increased risk of VV. The effect of prolonged standing posture at work on the development of VV remains unclear^11^.

According to pathophysiological mechanisms, prolonged standing contributes to the development of VV^12–14^. In healthy individuals, the deoxygenated blood in the lower leg is transported from the superficial into deep veins and back to the heart by skeletal muscle contractions and venous valves^12^. Even small leg movements are considered to ensure enough pumping action. Current knowledge suggests that prolonged standing without muscle activity increases the hydrostatic pressure, leading to enlargement of the veins^13^. As a consequence, over time, this increased hydrostatic pressure can lead to incompetent vein valves and walls. Insufficiently working vein valves provoke backward blood flows (reflux). A backward blood flow lasting for an exceptionally long time is defined as a pathological reflux^15^. In addition to the CEAP classification, duplex ultrasound is another diagnostic criterion for determining VV by assessing the backwards blood flow and reflux^16^. Progressively worsening insufficient vein valves provoke higher stages of CVI^12^. Similar to standing, extended sitting has been shown to impair muscle activity in lower limbs and increase the hydrostatic pressure, while physical activity stimulates the muscles’ pumping action and reduces the hydrostatic pressure^14^.

According to the European Agency for Safety and Health at Work (EU-OSHA) ^17^, every fifth worker in the European Union spends most of their working time in a standing posture. Prolonged standing means “standing continuously for more than one hour or standing for more than four hours” in total during one working shift^17^, p.6). Workers in occupations that require prolonged standing, e.g., nurses^18^ or hairdressers^19^, seem to experience an increased risk of developing VV. The latest results of a systematic review show that work-related aspects such as standing as a working posture and the sum of years of exposure to standing might be more important for contributing to the development of CVD compared to belonging to a specific occupational group^20^. Using accelerometer data of more than 80,000 adults from the UK Biobank, a recently published population cohort study investigated the association between stationary behaviour (standing and sitting) and the risk of VV and CVI^21^. Movement patterns suggested first a non-linear dose-risk association, second an increased risk of developing CVD when standing more than two hours per day, and third a risk increase of 22% per additional hour for individuals spending more than 12 h per day in a stationary posture^21^.

Until now, the effect of alternating between standing and sitting or even walking on the development of vein diseases, VV, and pathological reflux remain largely unknown. A first problem is that many studies do not differentiate between standing and walking when investigating the association with VV^22^. Second, most studies relied on self-reported exposure using questionnaires instead of device-based assessed data, e.g., wearables such as accelerometer^23^. Third, the vascular disorder-related outcome data are assessed very heterogeneously, e.g., via ultrasound measurement, self-reported questionnaires, or assessments through physicians. This makes comparisons of findings difficult^20,24^. Fourth, there is a lack of longitudinal studies evaluating the impact of the cumulative number of years of exposure on the development of CVD^24^. Although there are guidelines for improving ergonomics and working conditions in relation to constrained standing posture^25–27^, supporting evidence is limited, despite the pathophysiological rationale.

The primary aim of this study was to explore the cross-sectional association between standing time at one representative workday measured via accelerometer and the presence of VV and pathological reflux, respectively, among full-time working employees across diverse occupations. The secondary aim was to explore the dose-risk association between the long-term cumulative time spent standing within the reported occupation and the CVD status.

## Methods

### Study design

This epidemiological cross-sectional field study aimed to explore the risk of CVD and musculoskeletal issues associated with standing at work. The study design included two measurement days at the participant’s workplace. One day was aimed at tracking and documenting activity levels and body postures (standing, sitting, walking) over a 24-hour period. During the second day, a physical examination of the CVD status in lower legs, functional tests to evaluate the musculoskeletal system, and a self-reported survey were performed. This study project was conducted in accordance with the Declaration of Helsinki and approved by the ethics committee of the Medical Faculty, the University of Tübingen, and the University Hospital of Tübingen in September 2021 (No.: 571/2021BO2). Written informed consent was obtained from all participants included in the data analysis. The manuscript was prepared in accordance with the STROBE checklist^28^ and had been registered previously in the German Clinical Trials Register (DRKS-ID: DRKS00026975). In this manuscript, we report results related to the association of body postures during work hours and vein health. Remaining data analysis results are to be published in following manuscripts.

### Study population and recruitment

The sample size calculation was based on an expected Odds Ratio of 2.25 for the presence of CVD in the legs when comparing employees who stand for extremely long periods at work (>4 h) with those who do not (≤2 h). This expected Odds Ratio was derived from the findings of two previous cross-sectional studies investigating the risk of VV associated with standing at work for at least four hours using multiple logistic regression analysis, considering possible confounders^18,29^. The third exposure group (>2 but ≤4 h) was assigned the same sample size (*n* = 138). Statistical significance was set at two-sided p-value of < 0.05 with a power of 1-β ≥ 80%. An additional 15% of participants were aimed to be recruited to consider possible drop-outs, resulting in a total sample size of *n* = 476.

In terms of recruiting, initially, the target group comprised employees from trade and logistics companies, as workers in those occupational fields were previously reported to be exposed to prolonged standing^17^. We focused on recruiting volunteers. Therefore, managers were contacted for their support in the recruitment process by distributing an announcement along with participant information about study participation among corresponding employees. Kick-off meetings were organized on-site to explain the study’s objectives, in- and exclusion criteria, measurements to be conducted, as well as possible risks and benefits of participation. As not enough participants were successfully recruited in that first round, an internal email announcement was additionally distributed through the staff’s mail newsletter of the local University and University Hospital. Additionally, other local retailers, bakeries, pharmacies, and hairdressers were contacted using convenience sampling. In addition, newspaper announcements were published. Whether interested employees showed sufficient knowledge in the German language and met the inclusion criteria was checked through a telephone interview. Employees were eligible to take part if they were working in their profession for at least two years and a minimum 30 h on at least four days per week. During the recruiting phase, a minor change was made to the inclusion criteria, with the eligible age range adapted to 24 to 56 years from the original 25 to 55 years. Exclusion criteria included regular night shifts, current pregnancy, present coronary heart diseases or severe diseases of the cardiovascular system such as stroke or pulmonary embolism, polyneuropathy, blood coagulation disorders, previous surgeries of the spine or other spinal cord diseases, joint replacement in or paralysis of lower limbs. Participants were instructed to maintain their usual daily routine before the examination. The first participant was included in October 2021, while the last participant was included in April 2024. For participation on both measurement days, the participants received a financial compensation of 30€ as well as the results of their vein screening.

### Data collection and outcome measures

#### Accelerometer

Before the field measurements began, we checked that the accelerometer sensors could correctly detect body postures (standing, walking and sitting). Several participants wore the ActiGraph GT9X (ActiGraph, USA) to test the detection of the following:

- Walking and single steps with different stride lengths and directions.
- Sitting with stretched and bent legs.
- Sitting in different chair heights.

Overall, the results showed sufficiently good detection of the different body postures.

On the first day of the study, before starting work, the employees were given an accelerometer (ActiGraph GT9X Link, ActiGraph, USA) to record their physical activity pattern and body posture in real time and a heart rate sensor on the chest (H10, Polar, Finland) to measure the electrical activity of the heart for 24 h. The accelerometer was worn on the front of the thigh, on top of the trousers, and fastened with a belt. The beginning, break times, and end of working hours, as well as any interruptions in wearing time, e.g., for sleeping or taking a shower, were noted by the employees in a self-report activity diary during the 24 h-period. This allowed differentiation between work and leisure time when analyzing the data. All participants reported that the observed day was a usual work day. For this manuscript, only working time was considered.

Posture data and raw accelerometer data were stored using ‘ActiLife 6’ software (ActiGraph, USA). The raw 30 Hz accelerometer data were processed for quality checks using MATLAB version R2024a (MathWorks, Natick, USA). These checks included ensuring the sensor was worn correctly and that the data was consistent with the reported wearing times. The employee’s body posture (standing, walking or sitting) was determined every second by the original ActiGraph algorithm implemented in the firmware/ software. A so-called ‘period’ always starts and ends with the beginning of a walking phase (minimum three single steps). It can include sitting and/or standing in between. The mean period duration [s] is the average length of all periods on one day. Thus, a shorter mean period duration [s] reflects that an individual frequently changes body posture and walks more often.

#### Duplex ultrasound and CEAP

To describe the CVD-status, a CEAP classification as well as a duplex ultrasound measurement were conducted during the second measurement day. We used a real-time duplex ultrasound (DUS; B-mode, color and pulse-wave Doppler). The ultrasound assessments were conducted to evaluate the duration of the reflux in seconds. Reflux was assessed in seven vein segments in both legs according to Gloviczki et al.^16^:

- Common femoral vein proximal to the crosse (CFV),
- Femoral vein below the crosse (FV),
- Popliteal vein just above the popliteal fossa (PV),
- Great saphenous vein 5 cm below the crosse (GSV5),
- Great saphenous vein 15 cm below the crosse (GSV15),
- Small saphenous vein just below the popliteal fossa (SSV),
- And great saphenous vein at the lower leg below the popliteal fossa in the upper half of the calf (GSVC).

Whether to assess the left or right leg first was decided randomly prior to the measurement using an Excel worksheet and the function RAND(). A linear probe (7.5 MHz) of a mobile sonography device (Venue Go R3, GE Healthcare, US) was used for the measurements, according to Mendoza et al.^30^. Multiple measurement teams, consisting of two investigators each, were responsible for the measurements. The participant was standing barefoot on a wooden platform and wearing underpants or shorts, rolled up to the hip. The measurement protocol is described in detail in Soeder et al.^31^. The first investigator was responsible for guiding the ultrasound probe, whereas the second investigator performed the manual compression maneuver at midcalf-level.

The CEAP classification system was done according to the latest version published in 2004 by the American Venous Forum^4^. The first investigator was responsible for performing the CEAP classification. The focus of this study was on the C-aspect to describe the visible symptoms a participant might have depending on the presence and severity of CVD:

- C_0_: No visible or palpable signs of venous disease.
- C_1_: Telangiectasies or reticular veins.
- C_2_: Varicose veins; distinguished from reticular veins by a diameter of ≥ 3 mm.
- C_3_: Edema.
- C_4a_: Skin changes such as pigmentation or eczema.
- C_4b_: Skin changes such as lipodermatosclerosis or atrophy blanche.
- C_5_: Healed venous ulcer.
- C_6_: Active venous ulcer.

Etiology, anatomy, and pathophysiology are the three remaining aspects of the CEAP classification. Possible causes of existing venous disease (etiology) were not assessed. Aspects of anatomy (which veins are affected) and pathophysiology (duration of reflux only) were investigated using DUS as explained above.

### Self-reported questionnaire

During the second measurement day, the participants were asked to answer a paper-based questionnaire. The survey mainly included items and scales from prevailing and validated surveys. The following items were covered:

- Socio-demographics: age (complete years), gender (male, female, diverse), education (categorized as primary, secondary, or tertiary according to Lechert et al.^32^).
- Lifestyle factors: body height and weight to calculate the body mass index (BMI), smoking habit (currently, ex-smoker, non-smoker according to Zeiher et al.^33^, previous pregnancies (yes, no) and number of pregnancies (only for women), type and duration of physical activity in leisure time^34^.
- Perceived musculoskeletal demands in the workplace: physical demands in the workplace (relevant selection of items of Slesina^35^, frequency, severity of discomfort, and impairment of musculoskeletal discomfort in the lower back, legs, and feet within the last four weeks and twelve months according to the Cornell musculoskeletal discomfort questionnaire^36–39^.
- Psychosocial demands at work in terms of perceived social support in the workplace, job insecurity of the workplace, quantitative demands, influence at work, and job satisfaction according to Nübling et al.^40^.
- Occupational characteristics: self-reported occupational activity (free text), number of years with the current employer (complete years and months), number of years with the same occupational activity (complete years and months), working hours per week and day (free text), work ability index (WAI)^41^ using the first item^42,43^: ‘Current work ability compared with the lifetime best’ (rated from ‘completely unable to work’ (0) to ’currently best working ability’ (10)) and the sixth item^44^: ‘Own prognosis of work ability two years from now based on the current health state’ (three response options: hardly able to work, not sure, almost certain work ability).
- CVD predisposition: diseases in family history and possible indications of venous diseases similar to a questionnaire widely used in clinical practice in Germany according to Stücker et al.^45^.

### Statistical analysis

The study population’s characteristics are presented with number to treat, mean ± SD, and median [min; max] for continuous measures, whereas absolute and relative frequencies are shown for categorical variables.

The outcome variables were defined and analyzed as follows. The presence of VV (primary outcome) was handled as a binary variable (0 = no; 1 = yes), while VV here is defined as ≥C_2_. Reflux duration per vein segment (secondary outcome) was assessed by two independent raters via visual inspection of DUS-images showing the complete reflux curve. The x-axis represents time (in s) and the y-axis velocity (in cm/s). The curve below zero indicates a blood flow toward the heart (provocation manoeuver), above zero indicates backward flow. The end of reflux was marked by curve interruption or baseline intersection. The DUS-images were analyzed at a later stage, when all measurements had been completed, by two raters independently. The mean of both raters’ evaluated reflux time (in s to two decimals) was taken for analysis. This procedure should ensure the measurements’ reliability and objectivity following Soeder et al.^31^. Reflux duration lasting > 0.5s for superficial veins or > 1.0s for deep veins was considered pathological^16^ and then transferred into a binary variable (0 = no; 1 = yes).

The standing-related exposure variables were operationalized and analyzed as follows. The time spent sitting, standing, and walking during one workday is presented as a percentage of the overall workday. To investigate the association between standing duration per day (exposure variable) and CVD, standing duration was handled as a categorical variable (≤2 h standing, >2 to ≤4 h standing, >4 h standing per day) based on the accelerometer data, similar to Yun et al.^18^. Inferential statistics using the Kruskal-Wallis test were applied to compare those subgroups and explore whether observed differences between subgroups are relevant for later interpretation. If this were the case, further exploratory analyses, such as possible age-related effects, were carried out using the accelerometer data to investigate additional movement patterns in the data. To investigate the association between long-term cumulative occupational standing time and CVD, standing duration was considered as a continuous variable. This variable was derived by multiplying the number of months participants reported working in the same occupational activity by four (weeks per month), by their average working hours per week, and by the proportion of daily working time spent standing.

Additionally, for each independent variable collected via the previously described questionnaire, its role as a potential confounder or moderator for vein health was determined based on prior empirical findings^18,29^. This manuscript solely focuses on vein health. Relevant confounders for the multiple logistic regression models were selected from univariate logistic regressions (*p* < 0.05). A forward-backward selection approach was then applied to develop the final multiple logistic regression models. We are examining four different associations in total:

- Standing duration per day (≤ 2 h standing, > 2 to ≤4 h standing, >4 h standing per day) ~ ‘presence of varicose vein’ (0 = no; 1 = yes).
- Standing duration per day (≤ 2 h standing, > 2 to ≤4 h standing, >4 h standing per day) ~ ‘pathological reflux in at least one vein segment’ (0 = no; 1 = yes).
- Long-term cumulative occupational standing time (in h) ~ ‘presence of varicose vein’ (0 = no; 1 = yes).
- Long-term cumulative occupational standing time (in h) ~ ‘pathological reflux in at least one vein segment’ (0 = no; 1 = yes)’.

Regarding the results of the model-building approach, five models are presented for each association in the results section: null model (only intercept), model 1 includes only the standing duration as a predictor, model 2 shows the forward-backward generated multiple model, model 3 is the forward-backward generated multiple model with standing duration as additional predictor, and model 4 includes all predictors for comparison. In R, the step() function was used, having specified the direction of stepwise selection as ‘both’  (backward-forward) and starting with the empty model. In this approach, the model is optimized by removing insignificant variables and adding them again if they have then become statistically significant. Odds ratios (OR), p-values, and 95% confidence intervals (CI) are provided. The model fit was evaluated using Akaike information criterion (AIC), area under the curve (AUC; 0.5 = random, 1.0 = perfect accuracy), and Nagelkerkes Pseudo R^2^ (explained percentage of the total variance)^46,47^. Missing values were not imputed due to their low number. Participants of diverse gender were excluded from multiple analyses due to the low number. The models were computed again only on the subsample of women to consider the risk of previous pregnancies for VV. For an additional sensitivity analysis, the number of pathological reflux in vein segments per individual was treated as a numeric variable. Those results are presented in the Supplement.

Statistical significance was set at two-sided p-value of < 0.05. Statistical analyses were conducted using R version 4.4.3^48^. The package ggplot2 was used for the visualization of results. All packages used for statistical analyses are available on CRAN (Comprehensive R Archive Network, https://cran.r-project.org/web/packages/).

## Results

### Study participants

198 employees (*n* = 116 (59%) women) with a mean age of 40 ± 9 years from various departments and company sites of 70 companies out of the following industry sectors were included: logistics, trade, retail, manufacturing, healthcare, research, and service. Participants were working an average of 8.1 ± 1.1 h per day. 47 out of 198 (23.7%) participants were described with VV (19.8% of all men, 25.9% of all women). A pathological reflux was found in 74 (38%) participants (37.1% of all men, 38.3% of all women); among these, 36 (48.6%) participants had pathological reflux in more than one vein segment. The maximum reflux duration per individual did not differ significantly across CEAP classifications (Kruskal-Wallis Test: chi-squared = 131, p-value = 0.557). Nor were higher CEAP classes statistically significantly associated with longer reflux durations (Spearman: *p* = 0.065, rho = 0.13). The characteristics of the study population are shown in more detail in Table 1. The CONSORT flowchart is attached in the Supplement Figure S1.

**Table 1:** Characteristics of the study population (*N* = 198) and comparison of sociodemographic data between participants with and without varicose veins.

Characteristic	Overall, *N* = 198			No VV or CVI, *N* = 151			With VV or CVI, *N* = 47			*p*-value
	m (SD)	md [min; max]	*n* (%)	m (SD)	md [min; max]	*n* (%)	m (SD)	md [min; max]	*n* (%)	
Age (in years)	40 (9)	40 [24; 56]	198 (100)	39 (9)	37 [24; 56]	151 (100)	46 (7)	47 [26; 56]	47 (100)	*< 0.001*
18–29			29 (15)			27 (18)			2 (4)	
30–39			66 (33)			61 (40)			5 (11)	
40–49			63 (32)			38 (25)			25 (53)	
50–59			40 (20)			25 (17)			15 (32)	
Gender			198 (100)			151 (100)			47 (100)	*0.14*
Women			116 (59)			86 (57)			30 (64)	
Men			81 (41)			65 (43)			16 (34)	
Diverse			1 (0)			0 (0)			1 (2)	
BMI (in kg/m^2^)	26.0 (4.2)	25.3 [17.5; 39.2]	198 (100)	25.6 (4.0)	25.2 [17.5; 37.3]	151 (100)	27.3 (4.8)	26.2 [17.6; 39.2]	47 (100)	*0.036*
17-18.4 (underweight)			4 (2)			3 (2)			1 (2)	
18.5–24.9 (normal)			86 (43)			69 (45)			17 (36)	
25-29.9 (overweight)			75 (38)			57 (38)			18 (38)	
30-34.9 (adiposity I)			23 (12)			16 (11)			7 (15)	
35-39.9 (adiposity II)			10 (5)			6 (4)			4 (9)	
Education			198 (100)			151 (100)			47 (100)	*0.6*
Primary			50 (25)			38 (25)			12 (26)	
Secondary			118 (60)			88 (58)			30 (64)	
Tertiary			30 (15)			25 (17)			5 (10)	
Smoking habit			198 (100)			151 (100)			47 (100)	*0.5*
Current smoker			64 (32)			52 (34)			12 (25)	
Ex-smoker			50 (25)			37 (25)			13 (28)	
Non-smoker			84 (43)			62 (41)			22 (47)	
Previous pregnancy (only women)			115 (98)			85 (56)			30 (64)	*< 0.001*
Yes			62 (54)			38 (45)			24 (80)	
No			53 (46)			47 (55)			6 (20)	
Family history: varicose veins in parents			195			149 (99)			42 (93)	*0.3*
No			83 (42)			66 (44)			17 (36)	
One parent			101 (52)			76 (51)			25 (53)	
Both parents			12 (6)			7 (5)			5 (11)	
Tenure in same occupational activity with same employer (in months)	131 (97)	115 [2; 444]	198 (100)	121 (90)	91 [2; 383]	151 (100)	162 (113)	144 [6; 444]	47 (100)	*0.026*
Long-term cumulative occupational standing time (in h)	10,145 (8,396)	7,893 [262; 40,365]	173 (87)	9,483 (7,459)	7,947 [262; 40,365]	133 (88)	12,345 (10,777)	7,640 [455; 38,725]	40 (85)	*0.3*
Standing duration per day			198 (100)			151 (100)			47 (100)	*0.012*
≤2 h			36 (18)			26 (17)			10 (21)	
>2 h ≤4 h			91 (46)			78 (52)			13 (28)	
>4 h			71 (36)			47 (31)			24 (51)	
CEAP classification			198 (100)			151 (100)			47 (100)	*> 0.001*
C0			32 (16)			32 (21)			0 (0)	
C1			119 (60)			119 (79)			0 (0)	
C2			40 (20)			0 (0)			40 (85)	
C3			6 (3.0)			0 (0)			6 (13)	
C4a			1 (0.5)			0 (0)			1 (2.1)	
Pathological reflux			195 (98)			148 (98)			*47 (100)*	*0.002*
Yes			74 (38)			47 (32)			27 (51)	
No			121 (62)			101 (68)			23 (49)	
Number of veins with pathological reflux			195 (98)			148 (98)			*47 (100)*	*0.005*
0			121 (62)			101 (68)			20 (43)	
1			38 (19)			25 (17)			13 (28)	
2			20 (10)			14 (9.5)			6 (13)	
3			4 (2.0)			1 (0.7)			3 (6)	
4			9 (4.5)			5 (3.5)			4 (8)	
5			2 (1.0)			2 (1.3)			0 (0)	
7			1 (0.5)			0 (0)			1 (2)	

m = mean; sd = standard deviation; md = median; min = minimum; max = maximum; n = absolute numbers for categorical variables or number to treat for continuous variables; % = relative frequency; VV = varicose veins; CVI = chronic venous insufficiency (≥ C3 according to CEAP classification).

### Inferential statistics to compare subgroups with different amounts of wearable-based assessed standing exposure on one working day

**Fig. 1:**
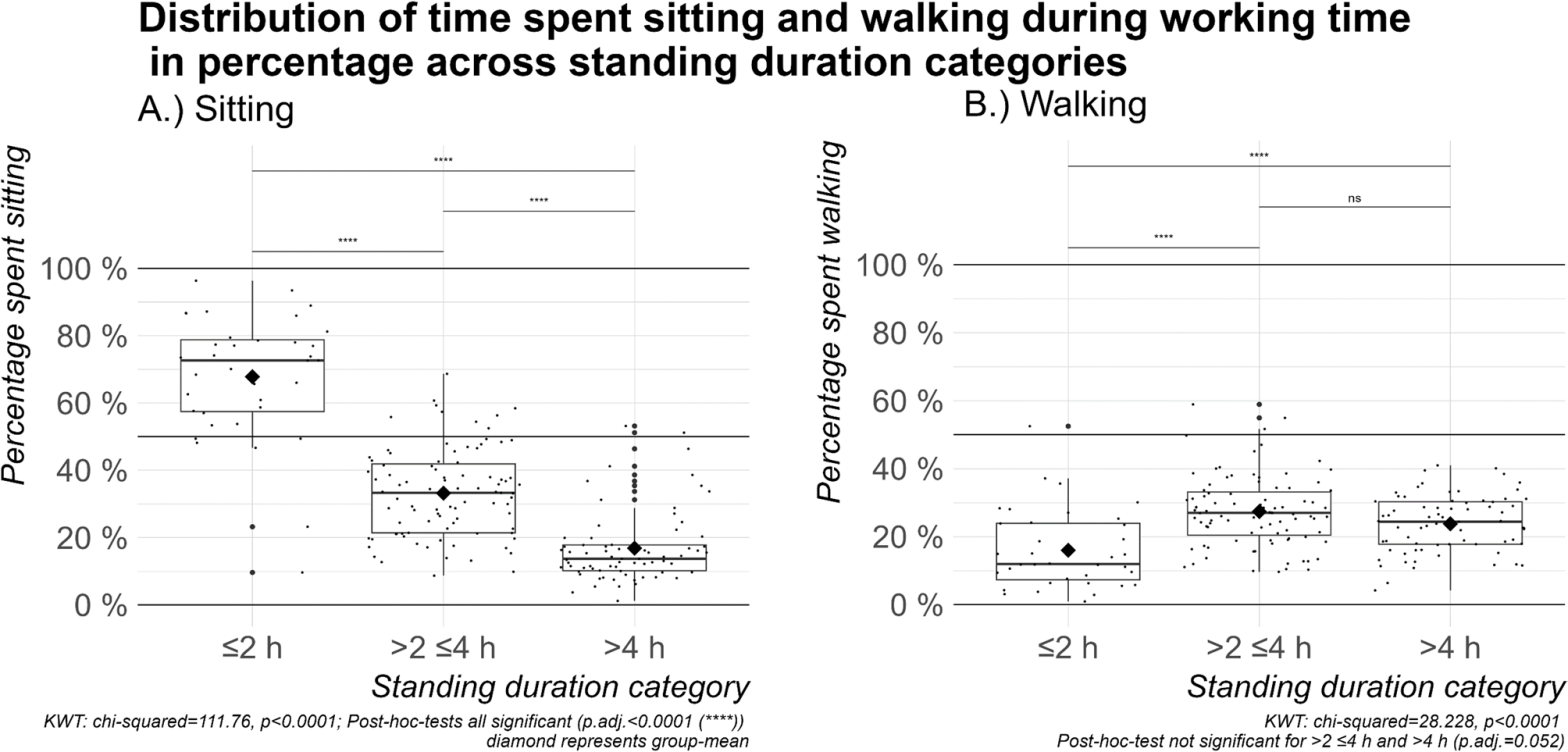
Distribution of the percentage of time spent (**A**) sitting and (**B**) walking during a working day across standing duration categories visualized using boxplots.

According to the wearable-based measurements, out of all participants, 18% (*n* = 36) stood ≤2 h, 46% (*n* = 91) >2 but ≤4 h, and 36% (*n* = 71) >4 h per workday. Figure 1 visualizes the differences in the percentage of time of a full working day spent sitting or walking among the three standing duration categories. Those employees standing ≤2 h sat for an average of 340 ± 126 min of the working day, while those standing >2 but ≤4 h sat 160 ± 77 min, and those standing >4 h sat 90 ± 69 min. Those standing ≤2 h walked in average 73 ± 47 min during the workday, while those standing >2 but ≤4 h walked 129 ± 54 min, and those standing >4 h walked 120 ± 43 min. The percentage spent sitting decreased statistically significantly with an increasing percentage spent standing (Kruskal-Wallis test: chi-squared = 111.76, *p* < 0.0001). In contrast, the percentage of time spent walking showed less pronounced differences across the three groups. There was no significant difference in the time spent walking when standing >2 but ≤4 h or >4 h (KWT: chi-squared = 28.228, *p* < 0.0001; post-hoc-test >2 but ≤4 h or >4 h: p.adj.=0.052).

### Association between standing duration per day and varicose veins or pathological reflux

Presence of VV: the univariate binary logistic regression analysis revealed no statistically significant association of standing duration per day with the presence of VV (0 = no; 1 = yes). Compared to standing less or equal to two hours per day, participants standing >2 but ≤4 h per day experienced a 0.43 times lower odd (*p* = 0.080 (95% CI=[0.17;1.24]), while those participants standing >4 h had 1.33 times the odds (*p* = 0.53, 95% CI=[0.56;3.29]; *n* = 198) to show VV. Table S1 in the Supplement presents the univariate binary logistic regression analyses results for all in this study included possible predictor variables with the presence of VV. The correlation between age and the number of years with the current employer was *r* = 0.58, which is why we excluded the latter as a possible confounder variable. Multiple binary logistic regression analysis was performed to evaluate possible factors, primarily standing duration per day, associated with the outcome ‘presence of varicose veins’, Table 2. The interaction effect between age and standing duration per day was not significant. Model 3 showed a significantly improved fit over the null model (X^2^(2) = 32.078; *p* < 0.001) and model 1 (X^2^(2) = 24.22; *p* < 0.001) but not over model 2 and 4. All models included *n* = 195 participants. Model 2 and Model 3 show a moderate model fit (AUC), and the explained variance in the outcome is moderate (R^2^). Having had a previous pregnancy as a risk factor for developing VV was tested in the subsample of women (*n* = 115) but did not show any statistical significance in the multiple model with age and standing duration per day, see Table S2 in the Supplement.

**Table 2:** Multiple logistic regression analysis to explore possible risk factors, primarily standing duration per day, for varicose veins (0 = no; 1 = yes), *N* = 195.

	Model 1				Model 2 (forward-backward-approach)				Model 3				Model 4			
	OR	*p*	lCI	uCI	OR	*p*	lCI	uCI	OR	*p*	lCI	uCI	OR	*p*	lCI	uCI
**Intercept**	**0.385**	**0.010**	**0.177**	**0.773**	**0.003**	**0.000**	**0.000**	**0.019**	**0.005**	**0.000**	**0.000**	**0.038**	**0.001**	**0.002**	**0.000**	**0.062**
Standing duration per day (ref = ≤ 2 h) >2 h ≤4 h >4 h	0.445	0.090	0.174	1.154					0.433	0.101	0.159	1.195	0.374	0.115	0.110	1.294
	1.272	0.593	0.534	3.171					0.795	0.650	0.294	2.174	0.717	0.575	0.222	2.312
**Age (years)**					**1.110**	**0.000**	**1.064**	**1.164**	**1.105**	**0.000**	**1.058**	**1.159**	**1.099**	**0.001**	**1.049**	**1.156**
Mean period duration [s]													1.002	0.619	0.995	1.008
Gender (ref = women)																
Men													0.629	0.264	0.273	1.404
BMI (in kg/m2)													1.067	0.188	0.970	1.176
Education (ref = primary)																
Secondary													1.494	0.409	0.585	3.990
Tertiary													0.850	0.831	0.180	3.655
Work ability item 1													1.030	0.827	0.794	1.352
**Family history: varicose veins in parents (ref = No)**																
**One parent**					1.581	0.237	0.746	3.436	1.560	0.256	0.730	3.419	1.428	0.379	0.649	3.209
**Both**					**4.451**	**0.040**	**1.043**	**18.797**	4.049	0.066	0.889	18.277	3.551	0.127	0.677	18.273
Smoking habit (ref = non-smoker)																
Current													1.095	0.851	0.422	2.819
Ex-smoker													0.817	0.684	0.303	2.138
AIC	214.02				192.33				192.98				203.27			
AUC	0.62				0.75				0.77				0.78			
R^2^	0.06				0.21				0.23				0.27			

lCI = lower CI; uCI = upper CI; ref = reference category; AIC = Akaike Information Criterion; AUC = Area under the Curve; R^2^ calculated using the pseudo R^2^ by Nagelkerke (1 = best fit); statistically significant results are indicated in bold (*p* < 0.05).

Presence of a pathological reflux: The univariate binary logistic regression analysis revealed no statistically significant association of standing duration per day with the ‘presence of a pathological reflux in at least one vein segment’ (0 = no; 1 = yes). Compared to standing less or equal to than two hours per day, participants standing >2 but ≤4 h per day experienced a 0.77 times lower odd (*p* = 0.52 (95% CI=[0.35;1.73]), while those participants standing >4 h had a 0.79 times lower odd (*p* = 0.57 (95% CI=[0.35;1.82]; *n* = 195) to show a pathological reflux. Table S3 in the Supplement presents the univariate binary logistic regression analyses results for all in this study included possible predictor variables with the presence of a pathological reflux. Multiple binary logistic regression analysis was performed to evaluate possible factors associated with the outcome ‘presence of a pathological reflux in at least one vein segment’, see Table 3. Model 3 showed a significantly improved fit over the null model (X^2^(2) = 14.374; *p* = 0.045) and model 1 (X^2^(2) = 13.87; *p* = 0.016) but not over model 2 and 4. All models included *n* = 193 participants. Model 2, 3, and 4 show a moderate model fit (AUC) but the explained variance in the outcome is low (R^2^). The interaction effect between age and standing duration was not statistically significant. Having had a previous pregnancy as a risk factor for developing a pathological reflux was tested in the subsample of women (*n* = 113) and did not show statistical significance in the forward-backward generated multiple model with age and standing duration, see Table S4 in the Supplement.

Table S5 in the Supplement presents the univariate linear regression analyses results for all in this study included possible predictor variables with the presence of a pathological reflux in one or more vein segments as a numerical outcome (min: 0; max: 7). Table S6 in the Supplement presents the multiple linear regression analyses results. Findings were not different to the logistic regression results.

**Table 3:** Multiple logistic regression analysis to explore possible risk factors, primarily standing duration per day, for the presence of at least one pathological reflux (0 = no; 1 = yes), *N* = 193.

	Model 1				Model 2 (forward-backward-approach)				Model 3				Model 4			
	OR	*p*	lCI	uCI	OR	*p*	lCI	uCI	OR	*p*	lCI	uCI	OR	*p*	lCI	uCI
Intercept	0.750	0.400	0.377	1.458	0.104	0.003	0.022	0.445	0.112	0.011	0.020	0.583	0.468	0.664	0.015	14.539
Standing duration per day (ref = ≤ 2 h) >2 h ≤4 h >4 h	0.749	0.400	0.377	1.458					0.686	0.374	0.298	1.588	0.853	0.753	0.317	2.323
	0.806	0.610	0.352	1.859					0.704	0.443	0.285	1.736	0.861	0.767	0.317	2.338
Age (years)					1.031	0.079	0.997	1.067	1.033	0.071	0.998	1.070	1.038	0.054	1.000	1.079
Mean period duration [s]													1.001	0.699	0.996	1.006
Gender (ref = women)																
Men													0.889	0.731	0.450	1.739
BMI (in kg/m2)													0.962	0.348	0.886	1.042
Education (ref = primary)																
Secondary													0.864	0.721	0.388	1.943
Tertiary													1.273	0.694	0.379	4.279
Work ability item 1													0.902	0.358	0.721	1.122
Family history: varicose veins in parents (ref = no)																
One parent									1.156	0.652	0.616	2.185	1.221	0.547	0.640	2.354
Both									2.839	0.119	0.772	11.187	3.194	0.096	0.825	13.222
**Smoking habit (ref = non-smoker)**																
**Current**					**2.453**	**0.019**	**1.163**	**5.238**	**2.427**	**0.025**	**1.123**	**5.321**	**2.584**	**0.017**	**1.198**	**5.706**
**Ex-smoker**					**2.541**	**0.010**	**1.253**	**5.251**	**2.531**	**0.012**	**1.235**	**5.286**	**2.712**	**0.017**	**1.208**	**6.229**
AIC	261.4884				252.7402				257.6189				266.4116			
AUC	0.53				0.64				0.66				0.67			
R^2^	< 0.01				0.08				0.09				0.12			

lCI = lower CI; uCI = upper CI; ref = reference category; AIC = Akaike Information Criterion; AUC = Area under the Curve; R^2^ calculated using the pseudo R^2^ by Nagelkerke (1 = best fit); statistically significant results are indicated in bold (*p* < 0.05).

### Distribution of CVD across age groups and daily standing duration

**Fig. 2:**
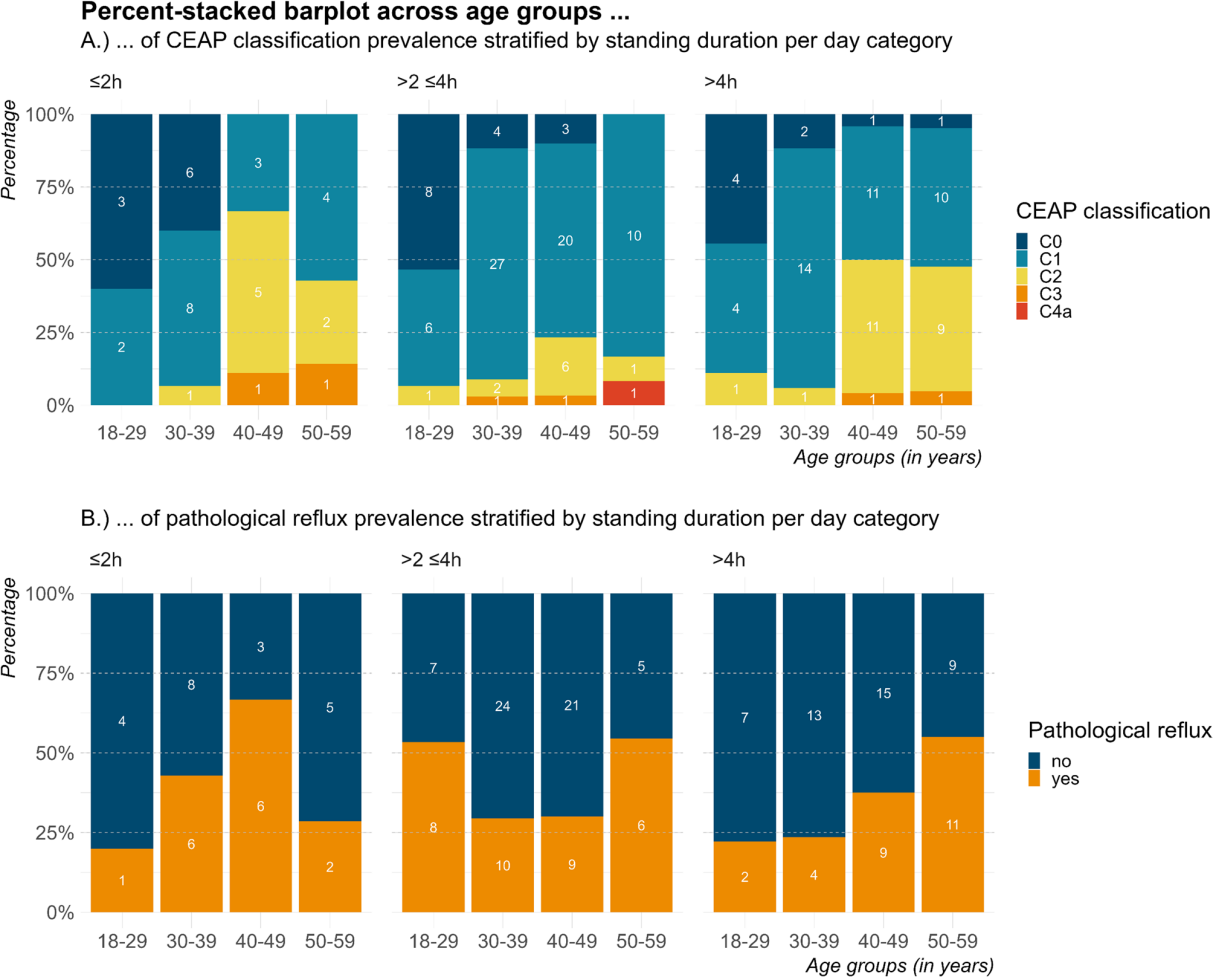
Distribution of (**A**) CEAP classifications and (**B**) presence of pathological reflux in at least one vein segment shown per age group stratified by standing duration per day category. White numbers reflect the absolute counts.

Multiple binary logistic regression analysis revealed age as an important predictor for VV and pathological reflux. Therefore, we exploratorily analyzed the movement patterns per day to visualize the variability of CVD among different age groups and standing duration categories. The aim was to see whether venous disease trends are consistent across all subgroups. The distribution of each CEAP classification and the presence of at least one pathological reflux, respectively, across age groups and stratified by standing duration per day category, is visualized in Fig. 2. CEAP classifications generally seem to increase with age across all standing duration categories. Overall, younger age seems to be linked to the lowest CEAP classifications. For participants over 40 years of age and standing for ≤2 h per day, a higher percentage of participants show a CEAP classification of ≥C_2_ than those of the same age standing >4 h per day (Fig. 2A). The risk of a pathological reflux seems to increase with age across all standing duration categories (Fig. 2B).

### Association between long-term cumulative occupational standing time and VV or pathological reflux, respectively

The univariate binary logistic regression analysis revealed no statistically significant association of long-term cumulative occupational standing time with the presence of VV (0 = no; 1 = yes; OR = 1.0; *p* = 0.067 (95% CI=[1.00;1.00]; *n* = 198). Table S1 in the Supplement presents the univariate binary logistic regression analyses results for all in this study included possible predictor variables with the presence of VV.

Table 4 shows the model summaries of the multiple binary logistic regression analysis with the outcome presence of VV. Model 3 showed a significantly improved fit over the null model (X^2^(2) = 19.95; *p* < 0.001) and model 1 (X^2^(2) = 17.792; *p* < 0.001) but not model 2 and 4. All models included *n* = 195 participants. Model 2 and Model 3 show a moderate model fit (AUC) but the explained variance in the outcome is low (R^2^). The correlation between age and long-term cumulative occupational standing time was *r* = 0.48; the interaction effect between age and standing duration was not significant. Having had a previous pregnancy as a risk factor for developing VV was tested in the subsample of women (*n* = 100), but did not show any statistical significance in the multiple model with age and long-term cumulative occupational standing time, see Table S7 in the Supplement.

**Table 4:** Multiple logistic regression analysis to explore possible risk factors, primarily long-term cumulative standing duration, for varicose veins (0 = no; 1 = yes), *N* = 195.

	Model 1				Model 2 (forward-backward-approach)				Model 3				Model 4			
	OR	*p*	lCI	uCI	OR	*p*	lCI	uCI	OR	*p*	lCI	uCI	OR	*p*	lCI	uCI
**Intercept**	**0.193**	**0.000**	**0.104**	**0.344**	**0.004**	**0.000**	**0.000**	**0.033**	**0.004**	**0.000**	**0.000**	**0.031**	**0.004**	**0.000**	**0.000**	**0.031**
Long-term cumulative occupational standing time [100 h]	1.004	0.080	0.999	1.008					0.999	0.645	0.994	1.004	0.999	0.645	0.994	1.004
**Age (years)**					**1.104**	**0.000**	**1.055**	**1.161**	**1.110**	**0.000**	**1.055**	**1.174**	**1.110**	**0.000**	**1.055**	**1.174**
Mean period duration [s]													1.003	0.354	0.997	1.008
Gender (ref = women)																
Men													0.879	0.760	0.376	2.007
BMI (in kg/m^2^)													1.051	0.333	0.950	1.163
Education (ref = primary)																
Secondary													1.241	0.686	0.443	3.660
Tertiary													0.943	0.939	0.197	4.116
Work ability item 1													1.055	0.729	0.784	1.452
Family history: varicose veins in parents (ref = no)																
One parent													1.355	0.478	0.589	3.194
Both													4.125	0.099	0.734	22.487
Smoking habit (ref = non-smoker)																
Current													1.336	0.563	0.497	3.589
Ex-smoker													1.097	0.862	0.377	3.130
AIC	172.6636				155.8901				157.6756				181.9757			
AUC	0.54				0.74				0.74				0.75			
R^2^	0.03				0.18				0.18				0.14			

lCI = lower CI; uCI = upper CI; ref = reference category; AIC = Akaike Information Criterion; AUC = Area under the Curve; R^2^ calculated using the pseudo R^2^ by Nagelkerke (1 = best fit). For a better visualization, the long-term cumulative occupational standing time was given in 100 h. Statistically significant results are indicated in bold (*p* < 0.05).

The univariate binary logistic regression analysis revealed no statistically significant association of the long-term cumulative occupational standing time with the presence of a pathological reflux (OR = 1.0; *p* = 0.719 (95-CI=[1.00;1.00]; *n* = 195). Table S3 in the Supplement presents the univariate binary logistic regression analyses results for all in this study included possible predictor variables with the presence of a pathological reflux. Table 5 shows the model summaries of the multiple binary logistic regression analysis with the outcome ‘presence of a pathological reflux in at least one vein segment’ (0 = no; 1 = yes). Model 3 showed a significantly improved fit over the null model (X^2^(2) = 21.015; *p* = 0.001) and model 1 (X^2^(2) = 20.542; *p* = 0.001) but not over model 2 and 4. All models included *n* = 193 participants. Model 2, 3, and 4 show a moderate model fit (AUC) but the explained variance in the outcome is low (R^2^). The correlation between age and standing duration was *r* = 0.49; the interaction effect between age and long-term cumulative occupational standing time and the main effect of long-term cumulative occupational standing time were significant, the main effect of age was not, see models three and four. Having had a previous pregnancy as a risk factor for developing a pathological reflux was tested in the subsample of women (*n* = 99) and did not show statistical significance in the forward-backward generated multiple model with age and standing duration, see Table S8.

**Table 5:** Multiple logistic regression analysis to explore possible risk factors, primarily long-term cumulative standing duration, for a pathological reflux (0 = no; 1 = yes), *N* = 193.

	Model 1				Model 2 (forward-backward-approach)				Model 3				Model 4			
	OR	*p*	lCI	uCI	OR	*p*	lCI	uCI	OR	*p*	lCI	uCI	OR	*p*	lCI	uCI
**Intercept**	**0.539**	**0.008**	**0.340**	**0.844**	0.104	0.762	-2.975	0.003	1.388	0.770	0.152	12.517	8.350	0.295	0.163	477.655
**Long-term cumulative occupational standing time [in 100 h]**	1.001	0.490	0.998	1.004					**0.964**	**0.005**	**0.938**	**0.988**	**0.965**	**0.010**	**0.937**	**0.990**
**Age (years)**					**1.031**	**0.017**	**1.759**	**0.079**	0.968	0.240	0.917	1.021	0.975	0.389	0.920	1.033
Long-term cumulative occupational standing time [h]* Age (years)									**1.001**	**0.004**	**1.000**	**1.001**	**1.001**	**0.007**	**1.000**	**1.001**
Mean period duration [s]													1.000	0.849	0.996	1.005
Gender (ref = women)																
Men													0.870	0.687	0.439	1.707
BMI (in kg/m2)													0.956	0.280	0.879	1.036
Education (ref = primary)																
Secondary													0.805	0.613	0.347	1.878
Tertiary													1.068	0.916	0.309	3.633
Work ability item 1													0.890	0.319	0.704	1.116
Family history: varicose veins in parents (ref = no)																
One parent													1.180	0.624	0.610	2.303
Both													3.038	0.119	0.761	13.073
**Smoking habit (ref = non-smoker)**																
**Current**					2.541	0.364	2.559	0.010	**2.885**	**0.006**	**1.376**	**6.223**	**2.791**	**0.014**	**1.247**	**6.430**
**Ex-smoker**					2.453	0.382	2.346	0.019	2.690	0.015	1.225	6.038	**2.955**	**0.012**	**1.283**	**7.021**
AIC	259.5204				252.7402				246.9788				257.8846			
AUC	0.47				0.65				0.69				0.71			
R^2^	<0.01				0.08				0.14				0.17			

lCI = lower CI; uCI = upper CI; ref = reference category; AIC = Akaike Information Criterion; AUC = Area under the Curve; R^2^ calculated using the pseudo R^2^ by Nagelkerke (1 = best fit); statistically significant results are indicated in bold (*p* < 0.05).

### Age-related patterns in the distribution of VV or pathological reflux

**Fig. 3:**
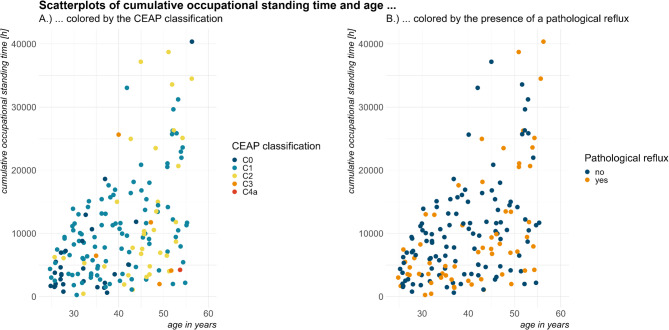
Distribution of long-term cumulative occupational standing time (h) across age (years), differentiated and colored by (**A**) CEAP classifications and (**B**) presence of at least one pathological reflux.

Age (in years) remained the only significant predictor of the ‘presence of varicose veins’ in the multiple binary logistic regression model, see Table 4. The interaction between age (in complete years) and long-term cumulative occupational standing time (in 100 h), but not the main effect of age, was statistically significant in the multiple binary logistic regression model for ‘presence of a pathological reflux in at least one vein segment’, see Table 5.  To investigate these relationships in more detail, we performed an exploratory analysis between age (in years) and long-term cumulative occupational standing time (in h) across the different CEAP classifications, see Fig. 3A, respectively, the presence of at least one pathological reflux, see Fig. 3B. While the severity of clinics according to CEAP classifications was shown to increase with age, no clear pattern was identified for the presence of pathological reflux.

## Discussion

The aim of this study was to explore the cross-sectional association between standing time at work measured via accelerometer and the presence of VV and duplex-ultrasound findings, such as the pathological reflux, respectively, among employees of diverse occupations. Neither the standing duration per day nor the long-term cumulative standing time within the same occupation were statistically significantly associated with the presence of VV (CEAP class ≥C_2_). However, increasing age and family history were statistically significantly associated with VV. Compared to participants standing ≤2 h per day, those standing >2 but ≤4 h per day had 0.45 times lower odds of experiencing VV, while those standing >4 h per day had 1.27 times higher odds. Although the OR of univariate analysis, see Table S2 in the Supplement, align with our expectations, the results were not statistically significant. The presence of a pathological reflux in at least one vein segment was positively statistically significantly associated with an interaction effect between long-term cumulative occupational standing time and age, as well as with the main effect of long-term cumulative occupational standing time. This was not the case for the categorical standing durations per day. Among the other explored possible predictor variables, smoking behavior was statistically significantly associated with the presence of a pathological reflux.

Out of 198 participants, 40 (20%) were described with VV, 7 (3.5%) with CVI, and 74 (38%) with a pathological reflux in at least one vein segment. The prevalence of VV ranges from 11.1 to 21.0% in the German and Western European society^1–3^ as described above. A prevalence of 35.3% of a pathological reflux was reported among the German population^15^. In a population-based observational study in German companies with more than 19,000 employees (age: 46 ± 10y; BMI: 26.1 ± 4.4), a prevalence rate of 3.7% was reported for CVI^1^. Thus, in comparison, the prevalence of VV and pathological reflux in our sample and the prevalence of CVI were similar to previous epidemiological studies. No participants were assigned to the classes C_4b_, C_5_, and C_6_ – more severe cases of CVI. Our finding that the CEAP classifications increase with age is similar to previous review’s findings^2^. According to previous studies^20,24^, prolonged standing time at work is associated with an increased risk of VV. In our sample, the exploratory analysis regarding the distribution of C in CEAP classifications across age groups stratified by standing duration category revealed that participants standing for >4 h per day showed the same or even lower CEAP classifications compared to those standing ≤2 h per day. This was particularly evident among participants over 40 years, which may indicate a healthy worker survivor effect in our sample. The healthy worker survivor effect describes a phenomenon of selection bias in the sense that healthy workers are more likely to remain in their workability compared to those with chronic diseases, poor health conditions, or other health issues^49^. In other words, in the long-term, those who remain in the workforce are usually the healthiest workers among their cohort. This effect often leads to bias, e.g., when computing prevalence rates in occupational populations or older age groups. As the healthy worker survivor effect may be present in our study population, the reported standing exposure-related risks for venous disorders might be underestimated.

In contrast to previous studies^18,29^, findings in our rather small sample did not confirm statistically significant associations between prolonged standing at work and the presence of VV or a pathological reflux, respectively. However, in univariate analysis a certain trend is present, as those participants standing >2 but ≤4 h per day had less chance of experiencing VV compared to participants standing ≤2 h per day, while those standing >4 h per day had increased chances of experiencing VV. The correlation between age and standing duration was moderate positive and the interaction effect between age and long-term cumulative occupational standing time was statistically significant. Even though we should interpret this finding with caution, it could suggest that the impact of standing depends on age. Exploratory analyses of available accelerometer data revealed that, when standing ≤2 h most participants sit extensively. While standing and sitting times differed across the three analyzed groups, walking time showed less variation. The available actigraphy data allowed an in-depth analysis of how movement patterns among different individuals were distributed across one working day, see Fig. S2 in the Supplement. Based on the described pathophysiological mechanisms and analyzed accelerometer data^14,21,50^, we would expect workers with high levels of lower leg muscle activation such as frequent walkers or workers with frequent transitions between standing, walking and sitting such as high movers, to show the lowest VV risk. In future work, it might therefore be more meaningful to compare activity patterns (sitting, standing, walking) rather than relying on standing categories based on recently used cut-off values. Following the suggestion of Ahmadi et al.^21^, that the dose-risk association between exposure duration and vein disease is non-linear, research on a new ‘risk index’ might help to consider that constrained standing increases vein pressure the most, sitting also but to a lesser extent, and only walking effectively reduces it.

### Strengths and limitations

A major strength is the objective assessment of exposure (accelerometer) and outcome (CEAP classifications). In comparison to previous studies evaluating the association between occupational standing duration and the presence of VV using self-reported data^18,29^, we relied on objectively measured standing, sitting, and walking time using accelerometer data. Another strength is that while we only assess accelerometer data of one typical working day, we additionally tried to cover the long-term cumulative duration of exposure working in the same occupation using self-reported data similar to Tabatabaeifar et al.^51^. Most previous studies lack to consider the number of years of exposure but focus on cross-sectional findings^24^. However, due to the cross-sectional design of our study, we cannot provide any information on causal relationships. Another strength is that we considered a variety of possible confounding parameters, gathered through a self-reported questionnaire.

A major limitation is that even though we put great effort in recruiting employees, the number of included participants was smaller than the a priori calculated sample size of 478 participants. One reason was the ongoing COVID-19 pandemic, during which companies refused external guests access to their company sites. As the recruited sample size did not reach the planned sample size, our study population might not be large enough to have a good chance of detecting a statistically significant effect of standing on VV. This is because the risk of a type II error is increased. However, the trends found can be used as a startpoint for further research. Because participants were mainly recruited via self-selection, a possible sample bias may limit the generalizability of our findings. As we relied on convenience sampling, we do not have any information about how many employees were contacted and how many decided to participate in our study. Thus, it is difficult to evaluate the representativeness of our study sample as well as potential reasons for nonresponse. Another limitation is that the accelerometer data was only collected on one single day.

Even though our participants categorized this day as representative of other working days in the current occupation, repeated measurements over several days should be carried out in future studies to obtain a more accurate assessment of working posture^52^. A common agreement on the definition of standing is currently not existing. For example, according to the EU-OSHA, “prolonged constrained or static standing also involves standing on the spot (movement restricted to a 20-cm radius) and not being able to obtain temporary relief by walking or sitting”^17^, p.6). In contrast, the German Federal Institute for Occupational Safety and Health developed a risk assessment using the key indicator method for awkward work-related body postures, where prolonged standing is defined as a static posture held for longer than one minute continuously or repeatedly held for longer than ten seconds^53^. These differences in definitions are critical when interpreting exposure data on work posture, as a few initial steps may have no physiological effect, whereas repeated interruptions might have. In addition, misclassifications of the raw data within the movement pattern are possible, even though the data was assessed using a gold standard, a thigh-worn wearable device. Another limitation is that our data mostly covered subjects exposed to prolonged sitting and/or standing. In a follow-up study, a more heterogeneous sample in terms of occupational walking time during the workday would help to consider the potential protective effect of walking on VV development.

### Future work

As our recruited sample size did not reach the planned sample size, the results should be interpreted with caution. Regarding future work, the exploratory analysis still revealed some methodological insights as well as practical considerations for future studies. Our sample is highly heterogeneous in terms of occupational groups and activities. This results in a large variety of movement patterns throughout one working day, making it difficult to only compare standing categories. As the effect of alternating between sitting, walking, and standing on developing VV remains largely unknown until now, it seems of high importance to compare activity patterns instead of categorical standing durations, e.g., by using accelerometer data. However, from a theoretical perspective, future work is essential to develop a definition of standing and an approach to differentiate various forms of standing by considering aspects such as posture and muscle activity. In our data, the reflux duration was not associated with clinical disease severity as presented in the CEAP classifications. Literature is rare on that topic and favors more into vein diameter measurement than reflux duration^54^. Leaving out DUS-measurements for reflux assessment would simplify the realization of future field studies on vein health in the workplace. Furthermore, there is a lack of longitudinal studies identifying the number of months and years of exposure, and their impact on the development of VV and CVI.

## Conclusion

This study examined the cross-sectional association between daily standing time at work, objectively assessed using accelerometer data, and the clinically assessed presence of VV via the C of CEAP classifications and pathological reflux via DUS, respectively, among employees across various occupations. Study results indicate that age, family history, and smoking had a significant impact on VV and pathological reflux. Study results did not confirm an association of the standing duration per day nor of long-term cumulative standing at work with CVD, despite the pathophysiological rationale for such an effect. Our findings indicate the advantage of objectively assessed data to capture the actual percentages spent standing, walking and sitting and thus consider the potential protective effect of walking. Future studies should benefit of this to better understand wearable-based assessed activity patterns and their impact on VV risk. This knowledge could then contribute to improving ergonomics and working conditions in terms of constrained static standing.

## Supplementary Information

Below is the link to the electronic supplementary material.

Supplementary Material 1

## Data Availability

The data are not publicly available due to data use restrictions contained in study participants’ information material. They are available on reasonable individual requests from the corresponding author Benjamin Steinhilber (Benjamin.Steinhilber@med.uni-tuebingen.de).
