# Supplementary material for: Accelerometer-based assessment of occupational standing time and its association with venous disorders – results of a cross-sectional field study
Source: Sci Rep. 2026 Feb 23;16:7477. doi: 10.1038/s41598-026-38327-8 (PMC12929726; doi:10.1038/s41598-026-38327-8)
Supplement: Supplementary file 1 — Supplementary Material 1 [file 41598_2026_38327_MOESM1_ESM.pdf]

Supplementary Information File

Figure S1: CONSORT flowchart

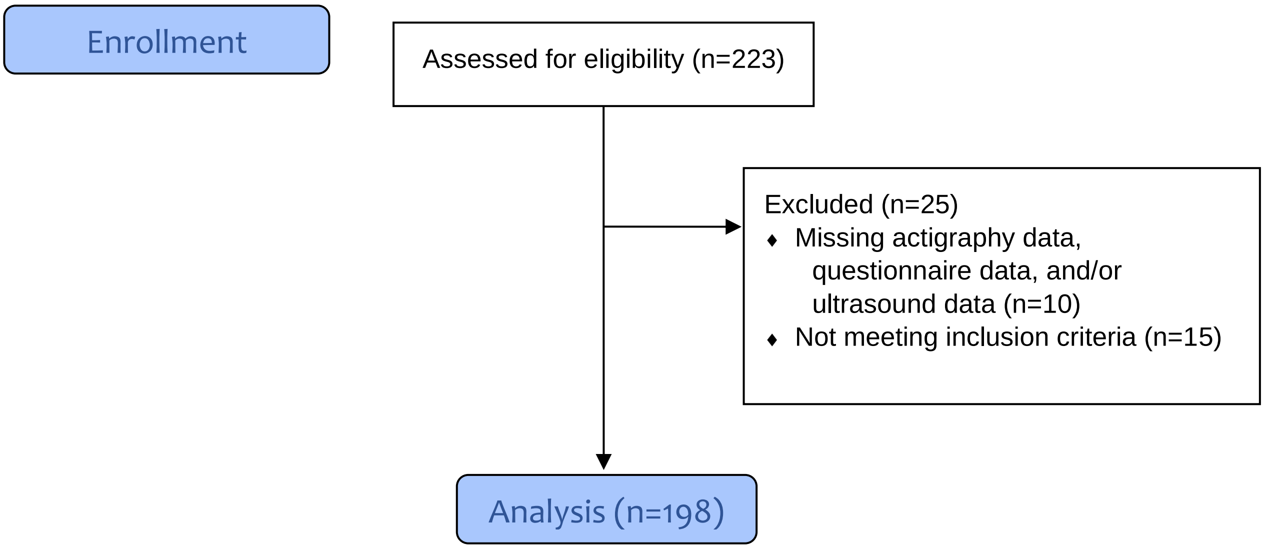

Supplementary Information File

Table S1: Presence of varicose veins (no=0; yes=1): results of analyses of all in this study included possible predictor variables using univariate binary logistic regression

Variable	statistics			
	OR	*p-value*	*Lower 95%-CI*	*Upper 95%-CI*
Standing duration per day [in h]	1.223	0.084	0.976	1.544
Standing duration per day (ref= ≤2h)				
>2h ≤4h	0.432	0.080	0.170	1.124
>4h	1.328	0.528	0.560	3.298
Long-term cumulative occupational standing time [in 100 h]	1.000	0.067	1.000	1.000
Mean period duration [in s]	1.001	0.623	0.997	1.005
**Age [in years]**	**1.101**	**0.000**	**1.057**	**1.151**
**Age groups [in years]**				
**30-39**	**1.107**	**0.907**	**0.223**	**8.063**
**40-49**	**8.882**	**0.005**	**2.367**	**58.133**
**50-59**	**8.100**	**0.009**	**2.018**	**54.745**
Gender (ref=women)				
men	0.706	0.320	0.349	1.388
**BMI**	**1.097**	**0.018**	**1.016**	**1.186**
BMI (ref=underweight)	0.739	0.799	0.088	15.451
Normal weight	0.895	0.925	0.107	18.731
Overweight	1.312	0.826	0.138	29.144
Adiposity I	2.000	0.600	0.174	48.880
Adiposity II				
Education (ref=primary)				
Seconday	1.080	0.846	0.508	2.397
Tertiary	0.633	0.440	0.183	1.938
Work ability item 1	1.007	0.956	0.804	1.281
Work ability in two years (ref=not sure) almost certain work ability	1.184	0.733	0.473	3.392
Family history: varicose veins in parents (ref=no)		0.493	0.639	2.602
One parent	1.277			
Both parents	2.773	0.114	0.742	9.814
Smoking habit (ref=non-smoker)				
Current	0.990	0.981	0.438	2.180
Ex-smoker	0.650	0.288	0.287	1.420
**Previous pregnancy (vs. no) (only women)**				
**yes**	**0.202**	**0.002**	**0.069**	**0.517**
statistically significant results are indicated in bold (p < 0.05).				

Table S2: Presence of varicose veins (no=0; yes=1): Logistic regression analyses computed only in the subsample of women (n=115) to consider the risk of previous pregnancies

Variable	*OR*	*p-value*	*Lower 95%-CI*	*Upper 95%-CI*
**Intercept**	**0.003**	**0.007**	**0.000**	**0.231**
Standing duration per day (ref= ≤2h)	0.321	0.138	0.070	1.486
>2h ≤4h	0.855	0.837	0.187	3.926
>4h				
**Age [in years]**	**1.106**	**0.004**	**1.036**	**1.191**
Family history: varicose veins in parents (ref=no)	1.542	0.429	0.537	4.663
One parent	6.208	0.101	0.756	63.547
Both parents				
Previous pregnancy (vs. no) (only women) yes	3.195	0.056	0.056	1.013
statistically significant results are indicated in bold (p < 0.05).				

Table S3: Presence of pathological reflux (no=0; yes=1): results of analyses of all in this study included possible predictor variables using univariate binary logistic regression

**Variable**	**Statistics**			
	OR	***p-value***	***Lower 95%-CI***	***Upper 95%-CI***
Standing duration per day [in h]	1.004	0.972	0.823	1.224
Standing duration per day (ref= ≤2h)<br>>2h ≤4h<br>>4h	0.772<br>0.788	0.523<br>0.572	0.349<br>0.345	1.726<br>1.815
Long-term cumulative occupational standing time [in 100 h]	1.000	0.719	1.000	1.000
Mean period duration [in s]	1.000	0.870	0.997	1.004
Age [in years]	1.025	0.128	0.993	1.059
Age groups [in years]<br>30-39<br>40-49<br>50-59	0.727<br>1.007<br>1.636	0.496<br>0.988<br>0.326	0.292<br>0.410<br>0.617	1.848<br>2.540<br>4.459
Gender (ref=women)<br>men	1.045	0.884	0.578	1.879
BMI (in kg/m2)	0.983	0.627	0.916	1.053
BMI (ref=underweight)<br>Normal weight<br>Overweight<br>Adiposity I<br>Adiposity II	2.143<br>1.723<br>1.600<br>1.286	0.517<br>0.645<br>0.704<br>0.852	0.262<br>0.209<br>0.171<br>0.103	44.304<br>35.781<br>35.327<br>32.061
Education (ref=primary)<br>Seconday<br>Tertiary	0.863<br>1.000	0.672<br>1.000	0.438<br>0.392	1.720<br>2.514
Work ability item 1	0.869	0.174	0.707	1.063
Work ability in two years (ref=not sure)<br>almost certain work ability	0.944	0.643	0.742	1.211
Family history: varicose veins in parents (ref=no)<br>One parent<br>Both parents	1.111<br>2.607	0.734<br>0.128	0.606<br>0.766	2.049<br>9.507
**Smoking habit (ref=non-smoker)**<br>**Current**<br>**Ex-smoker**	**2.453**<br>**2.365**	**0.018**<br>**0.015**	**1.171**<br>**1.187**	**5.203**<br>**4.785**
Previous pregnancy (vs. no) (only women)<br>yes	1.312	0.488	0.612	2.847
statistically significant results are indicated in bold (p < 0.05).				

Table S4: Presence of pathological reflux (no=0; yes=1): Logistic regression analyses computed only in the subsample of women (n=115) to consider the risk of previous pregnancies

**Variable**	***OR***	***p-value***	***Lower 95%-CI***	***Upper 95%-CI***
Intercept	0.209	0.227	0.015	2.538
Standing duration per day (ref= ≤2h)<br>*>2h ≤4h*<br>*>4h*	0.509<br>0.736	0.274<br>0.660	0.149<br>0.186	1.744<br>2.927
Age [in years]	1.011	0.696	0.956	1.070
**Family history: varicose veins in parents (ref=no)**<br>**One parent**<br>**Both parents**	**1.656**<br>**15.902**	**0.270**<br>**0.018**	**0.687**<br>**2.179**	**4.171**<br>**331.586**
Smoking habit (ref=non-smoker)<br>Current<br>Ex-smoker	2.173<br>2.537	0.169<br>0.074	0.721<br>0.920	6.694<br>7.215
Previous pregnancy (vs. no) (only women)<br>yes	1.318	0.584	0.493	3.621
statistically significant results are indicated in bold (p < 0.05).				

Supplementary Information File

Table S5: Presence of pathological reflux in one or more vein segments (ranging from 0-7): results of analyses of all in this study included possible predictor variables using univariate linear regression

Variable	Statistics			
	Regression Coef.	*Std error*	*t-value*	*p-value*
Standing duration per day [in h]	−0.003	0.05917086	-0.05871602	0.953
Standing duration per day (ref= ≤2h)<br>>2h ≤4h	−0.141	0.2414209	-0.5841485	0.560
>4h	−0.129	0.2508744	-0.5146319	0.607
Cumulative occupational standing time [in 100 h]	0.000	1.079778e-05	-0.2477625	0.805
Mean period duration [in s]	−0.001	0.001078302	-0.6212899	0.535
Age [in years]	0.013	0.009477396	1.3720618	0.172
Age groups [in years]<br>30-39	0.031	0.2725944	0.1130819	0.910
40-49	0.062	0.2745685	0.2252627	0.822
50-59	0.354	0.2984156	1.1873049	0.237
Gender (ref=women)<br>men	0.130	0.1773072	0.7335027	0.464
BMI (in kg/m2)	−0.007	0.02061529	-0.3627518	0.717
BMI (ref=underweight)<br>Normal weight	0.349	0.6265316	0.55677516	0.578
Overweight	0.153	0.6285703	0.24393984	0.808
Adiposity I	−0.065	0.6635733	-0.09828212	0.922
Adiposity II	0.400	0.7246611	0.55198217	0.582
Education (ref=primary)<br>Secondary	−0.293	0.2060103	-1.4216831	0.157
Tertiary	−0.153	0.2819419	-0.5438472	0.587
**Work ability item 1**	**−0.138**	**0.06036697**	**-2.281268**	**0.024**
Work ability in two years (ref=not sure)<br>almost certain work ability	−0.042	0.2486900	-0.1675446	0.867
Family history: varicose veins in parents (ref=no)<br>One parent	−0.103	0.1787194	-0.5786927	0.563
Both parents	0.253	0.3725586	0.6791201	0.498
**Smoking habit (ref=non-smoker)<br>Current**	**0.508**	**0.2151671**	**2.359185**	**0.019**
**Ex-smoker**	**0.438**	**0.1998708**	**2.192636**	**0.030**
Previous pregnancy (vs. no) (only women)<br>yes	−0.313	0.2214153	-1.414295	0.160
statistically significant results are indicated in bold (p < 0.05).				

Table S6: Presence of pathological reflux in one or more vein segments (ranging from 0-7): results of the multivariate linear regression analysis to explore possible risk factors, primarily standing duration per day

	Model 1				Model 2 (forward-backward-approach)				Model 3				Model 4			
	Coef	p	lCI	uCI	Coef	p	lCI	uCI	Coef	p	lCI	uCI	Coef	p	lCI	uCI
Intercept	0.857	0.205	4.178	0.000	0.809	0.686	1.179	0.240	0.927	0.705	1.314	0.191	1.779	0.964	1.844	0.067
Standing duration per day (ref=≤2h)	−0.194	0.242	-0.802	0.424					−0.186	0.237	-0.784	0.434	−0.167	0.281	-0.594	0.553
>2h ≤4h																
>4h	−0.133	0.252	-0.526	0.599					−0.101	0.249	-0.404	0.687	−0.100	0.279	-0.358	0.721
**Age (in years)**					**0.014**	**0.009**	**1.522**	**0.130**	**0.014**	**0.009**	**1.419**	**0.157**	**0.017**	**0.010**	**1.5958**	**0.112**
**Mean period duration [in s]**													**−0.001**	**0.002**	**-0.512**	**0.610**
Gender (ref=women)																
Men													0.002	0.189	0.009	0.992
BMI (in kg/m2)													−0.031	0.023	-1.364	0.174
Education (ref= primary)																
Secondary													−0.236	0.226	-1.043	0.299
Tertiary													0.034	0.342	0.100	0.920
Work ability item 1					−0.111	0.060	-1.838	0.068	−0.108	0.061	-1.783	0.076	−0.108	0.062	-1.738	0.084
Family history: varicose veins in parents (ref=no)																
One parent													−0.012	0.182	-0.068	0.946
Both													0.370	0.388	0.953	0.342
Smoking habit (ref=non-smoker)																
Current					0.398	0.201	1.976	0.050	0.411	0.203	2.025	0.044	0.357	0.215	1.656	0.099
Ex-smoker					0.494	0.213	2.321	0.021	0.507	0.219	2.320	0.021	0.538	0.227	2.371	0.019
AIC	627.4851				618.3910				621.7260				630.9438			
R^2	0.003				0.069				0.072				0.095			

lCI=lower CIM uCI=upper CI; AIC = Akaike Information Criterion; AUC = Area under the Curve; R2 calculated using the pseudo R2 by Nagelkerke (1= best fit); statistically significant results are indicated in bold (p < 0.05).

Table S7: Presence of varicose veins (no=0; yes=1): Logistic regression analyses computed only in the subsample of women (n=100) to consider the risk of previous pregnancies

**Variable**	***OR***	***p-value***	***Lower 95%-CI***	***Upper 95%-CI***
**Intercept**	**0.001**	**0.000**	**0.000**	**0.026**
Long-term cumulative occupational standing time [in 100 h]	**0.999**	**0.003**	**1.045**	**1.224**
Age [in years]	1.125	0.746	0.992	1.006
Previous pregnancy (vs. no) (only women) yes	1.993	0.265	0.610	7.185
statistically significant results are indicated in bold (p < 0.05).				

Table S8: Presence of pathological reflux (no=0; yes=1): Logistic regression analyses computed only in the subsample of women (n=99) to consider the risk of previous pregnancies

**Variable**	***OR***	***p-value***	***Lower 95%-CI***	***Upper 95%-CI***
Intercept	13.270	0.197	0.265	746.453
Long-term cumulative occupational standing time [in 100 h]	**0.999**	**0.006**	**0.999**	**1.000**
Age [in years]	0.903	0.063	0.807	1.003
Family history: varicose veins in parents (ref=no)<br>One parent	1.519	0.416	0.563	4.294
Both parents	7.028	0.123	0.764	164.554
**Smoking habit (ref=non-smoker)**<br>**Current**	**2.512**	**0.129**	**0.774**	**8.578**
**Ex-smoker**	**4.367**	**0.018**	**1.321**	**15.570**
Previous pregnancy (vs. no) (only women)<br>yes	2.304	0.195	0.678	8.695
**Long-term cumulative occupational standing time [h]* Age (years)**	**1.000**	**0.006**	**1.000**	**1.000**
statistically significant results are indicated in bold (p < 0.05).				

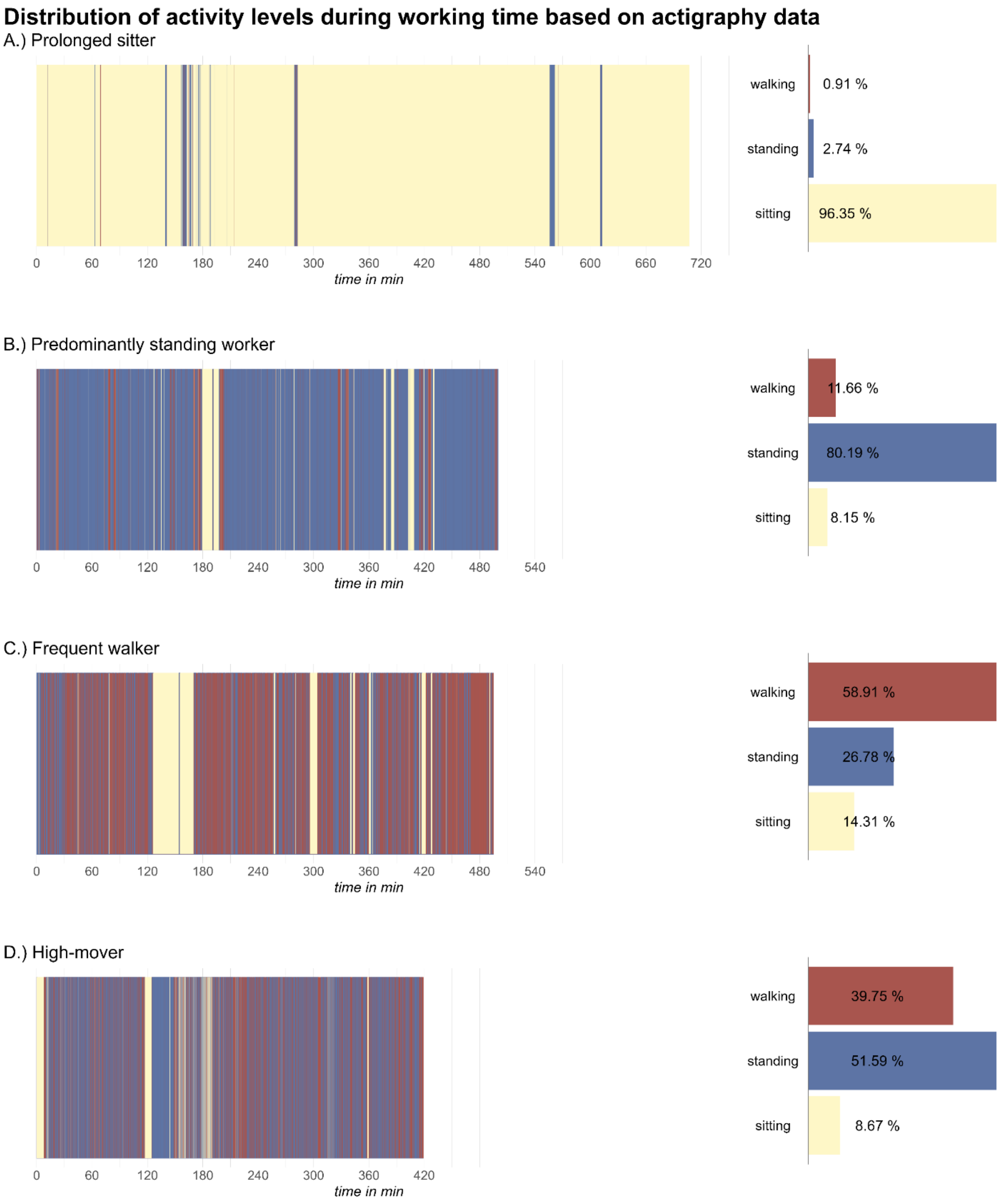

Fig. S2. Distribution of activity levels during working time compared between four individuals with extreme differences in movement patterns based on actigraphy data: prolonged sitter / ≤2h (A), predominantly standing worker / >4h (B), frequent walker (>2 but ≤4h) (C), and high-mover (>2 but ≤4h) (D).
